# Development and Prospective Applications of 3D Membranes as a Sensor for Monitoring and Inducing Tissue Regeneration

**DOI:** 10.3390/membranes13090802

**Published:** 2023-09-18

**Authors:** Hanning Wu, Jiawen Chen, Pengxiang Zhao, Mengyu Liu, Fei Xie, Xuemei Ma

**Affiliations:** Faculty of Environment and Life, Beijing University of Technology, Beijing 100124, Chinaxiefei990815@bjut.edu.cn (F.X.); xmma@bjut.edu.cn (X.M.)

**Keywords:** tissue regeneration, 3D printing, sensors, biomaterials, artificial membrane application

## Abstract

For decades, tissue regeneration has been a challenging issue in scientific modeling and human practices. Although many conventional therapies are already used to treat burns, muscle injuries, bone defects, and hair follicle injuries, there remains an urgent need for better healing effects in skin, bone, and other unique tissues. Recent advances in three-dimensional (3D) printing and real-time monitoring technologies have enabled the creation of tissue-like membranes and the provision of an appropriate microenvironment. Using tissue engineering methods incorporating 3D printing technologies and biomaterials for the extracellular matrix (ECM) containing scaffolds can be used to construct a precisely distributed artificial membrane. Moreover, advances in smart sensors have facilitated the development of tissue regeneration. Various smart sensors may monitor the recovery of the wound process in different aspects, and some may spontaneously give feedback to the wound sites by releasing biological factors. The combination of the detection of smart sensors and individualized membrane design in the healing process shows enormous potential for wound dressings. Here, we provide an overview of the advantages of 3D printing and conventional therapies in tissue engineering. We also shed light on different types of 3D printing technology, biomaterials, and sensors to describe effective methods for use in skin and other tissue regeneration, highlighting their strengths and limitations. Finally, we highlight the value of 3D bioengineered membranes in various fields, including the modeling of disease, organ-on-a-chip, and drug development.

## 1. Introduction

**Figure 1:**
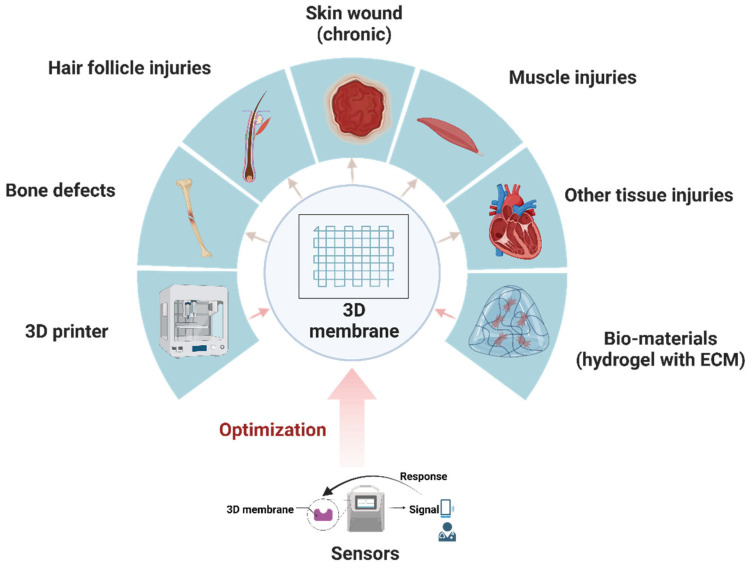
A concept map of sensor-binding 3D membranes. By optimizing 3D membrane with smart sensors, it may induce a better regeneration effect in various tissues. Created with BioRender.com (accessed on 21 August 2023).

Over the past decade, tissue regeneration has remained an important challenge to overcome. Tissue injuries, especially those of the skin and its appendages, muscles, and bones, are prevalent insults that disrupt homeostasis and give rise to common problems in clinical practice [1,2,3,4]. The defection of these tissues can severely affect the quality of human life, resulting in various inconveniences and social burdens [5]. Skin diseases, bone defects, and muscle abnormalities are all associated with chronic, recurrent processes that urgently require novel, efficient treatments [6,7]. Despite the development of some regenerative wound therapeutic strategies, including skin substitutes, hair transplantation, non-steroidal anti-inflammatory drugs, and traditional tissue engineering, these techniques remain unable to effectively deal with complex injuries, such as chronic wounds, severe burns, or the creation of full-thickness grafts [5,8,9,10,11,12]. In addition, some traditional regenerative therapies for inner tissues (e.g., bones and muscles), such as hydrogel dressings and bone graft surgery, have shown reduced superiority in terms of inherited muscle defects and new bone formation [13,14,15]. Moreover, the shortage of suitable organ donors, religious beliefs, and other factors make organ donation (e.g., heart and kidney donation) in short supply, placing pressure on organ repair and giving rise to a greater need for organ regeneration [16,17]. Hence, tissue engineering represents an ideal strategy to construct biological tissue substitutes, which can be used to regenerate or restore tissue defects or can be used as an in vitro device to temporarily replace organ functions and improve the quality of life [18,19]. Tissue engineering methods for tissue regeneration include three key elements: scaffolds, cells, and growth factors [20,21]. Tissue engineering scaffolds are the most basic structure of tissue engineering, and without these biological support materials, cells and growth factors cannot be placed, and tissues or organs cannot be generated. Various production techniques have been developed to develop a more stabilized and functionalized scaffold for tissue regeneration; these include electrospinning, phase separation, gas foaming, the porogen method, polymerization in solution, self-assembly, membrane lamination, freeze drying, and 3D printing [22]. Among these methods, 3D printing can effectively solve the plights of spatial positioning and structure scanning in constructing reliable and standardized tissue scaffolds [23,24,25,26,27]. Moreover, a series of electronic elements are integrated with tissue regenerative scaffolds to elicit and monitor the behavior of the regeneration process, and thus, promote the intelligence and responsiveness of 3D scaffolds [28,29,30]. These types of 3D scaffolds and other membrane-like artificial smart tissues for tissue regeneration are termed “3D regenerative membranes”. The concept map of the 3D regenerative membrane is shown in Figure 1.

This review describes tissue regeneration using sensor-binding 3D membranes, as well as the challenges in their clinical application. We also summarize the perspectives of 3D bioengineered membranes in various fields, including disease modeling [31,32], electronic skin [33], organ-on-a-chip [34], and drug development [35].

## 2. 3D Printing

3D printing, also known as additive manufacturing, uses computer-aided manufacturing (CAM) to build up the printing inks layer-by-layer per the data obtained from 3D digital models [36,37]. The process involves three core elements: a 3D printer, printing inks, and a computer-aided design (CAD)/CAM system. Recent developments have made it possible to use 3D printing in tissue engineering and regenerative medicine [38]. Compared with conventional methods, 3D printing provides benefits such as an individualized design, the integrated production of functionalized scaffolds, and the rapid creation of complex structures [39,40]. Advanced manufacturing of 3D membrane and sensors can use 3D printing in clinical research. In this section, we introduce several 3D printing methods as well as printing inks (especially biomaterials) and discuss the usages of 3D printing in the context of 3D membrane construction and the limitations of 3D membranes without sensors.

### 2.1. Approaches of 3D Printing

**Figure 2:**
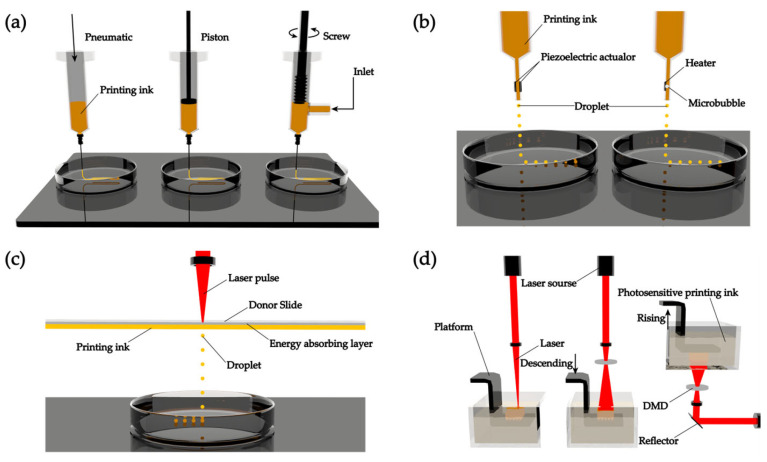
Various 3D printing technologies: (**a**) extrusion-based 3D printing; (**b**) droplet-based 3D printing; (**c**) laser-assisted 3D printing; and (**d**) stereolithography-based 3D printing.

Based on existing research, common 3D printing technologies can be roughly divided into four categories: extrusion-based 3D printing (Figure 2a) [41,42,43,44,45], droplet-based 3D printing (Figure 2b) [46,47,48], laser-assisted 3D printing (Figure 2c) [49,50,51,52], and stereolithography-based 3D printing (Figure 2d) [53,54]. Although each 3D printing technology has its advantages and limitations, they all contribute to the individualization and intelligent manufacturing of 3D membranes and sensors.

#### 2.1.1. Extrusion-Based 3D Printing

Extrusion-based 3D printing, as shown in Figure 2a, uses a pneumatic, piston, or screw to extrude the printing inks under constant pressure. By adjusting the environmental conditions, the printing inks form continuous microfilaments, which are then deposited layer-by-layer onto the printing platform [41]. This type of 3D printing technology is currently the most widely used and is suitable for printing large-scale complex structures as well as high-viscosity materials. However, as a disadvantage, this method has low accuracy (resolution > 100 μm) [44]. Therefore, extrusion-based 3D printing is suitable to obtain 3D membranes and sensors with large size and low accuracy requirements (resolution > 100 μm), rather than nano-precision sensors.

#### 2.1.2. Droplet-Based 3D printing

Droplet-based 3D printing, as shown in Figure 2b, generates microbubbles at the nozzle tip via piezoelectric or thermal energy, which in turn drives the printing inks’ droplets to be ejected and deposited layer-by-layer on the printing platform [46]. This technology has high printing accuracy, fast printing speed, and low printing cost. However, the prominent disadvantage of this technology is that the printing inks have low viscosity, and it is difficult to form complex 3D structures due to the challenges in obtaining the desired shapes [55]. Therefore, droplet-based 3D printing is suitable to obtain high-precision and small-sized sensors with multilayer high-precision 3D membranes. Zhang et al. [56] used droplet-based 3D printing to spray graphene oxide (GO) on the substrate to form a nano-precision, soft, ultra-thin, flexible, conductive, and biocompatible nano-heart patch for myocardial infarction (MI) repair. Similar to this research, it is an advanced application of droplet-based 3D printing to construct the nano-sensor module by printing highly conductive materials (e.g., GO and liquid metal).

#### 2.1.3. Laser-Assisted 3D Printing

Laser-assisted 3D printing, as shown in Figure 2c, uses laser pulses to create microbubbles in the printing inks’ layer; the printing inks’ droplets are propelled to be ejected and deposited layer-by-layer on the printing platform [40]. This method can avoid direct contact between the material and the nozzle, resulting in high viscosity of the printed material. However, the main disadvantage of this technology is the high cost [49]. Therefore, laser-assisted 3D printing is suitable for the molding of some printing inks with high requirements for the molding environment to expand the application of bioinks (containing viable cells) and some ink with harsh molding conditions in 3D membranes and sensor manufacturing.

#### 2.1.4. Stereolithography-Based 3D Printing

Stereolithography-based 3D printing, as shown in Figure 2d, is suitable for use with photo-crosslinking printing inks, in which selective curing is achieved by controlling visible or ultraviolet light. This technology has high printing accuracy, fast printing speed, and good quality on the vertical surface. However, laser irradiation and photoinitiator toxicity can cause damage to the cells. Moreover, the selection of the type of printing inks is limited [53]. In recent years, two-photon polymerization (TPP) [57] and microscale computed axial lithography (micro-CAL) [58] were developed on the basis of stereolithography-based 3D printing, which have provided new ideas for the rapid, refined, and integrated manufacturing of nano-scale sensors and high-precision 3D membranes.

### 2.2. Printing Inks

Printing inks, typically composed of biomaterials, are an important component of 3D printing for 3D regenerative membranes. Suitable printing inks allow the 3D regenerative membranes to better satisfy the design requirements of tissue engineering. The existing biomaterials used in printing inks can be divided into animal-sourced natural ECM materials, non-animal-derived natural hydrogels, and synthetic hydrogels [59]. Table 1 summarizes the crosslinking characteristics and the applicable 3D printing methods of the various types of biomaterials [60,61,62,63].

### 2.3. 3D Membranes in Different Tissues and Organs

Many 3D membranes are constructed using 3D printing owing to its suitability for manufacturing complex structures with multiple layers. Common regenerative 3D printing membranes in different tissues and organs include skin membranes, serosal membranes, tubular tissue membranes, and connective tissue membranes [85]. A summary of the 3D printing manufacturing processes for various types of membranes is provided in the following text.

#### 2.3.1. Skin Membranes

Skin membranes [86,87] are suitable for problems with tissue regeneration related to skin and its appendages, such as acute or chronic wound healing. The constructed skin membrane often has a multilayered structure, including an epidermal layer, a dermal layer, a subcutaneous tissue layer, and a skin appendage layer [88]. Currently, to construct a fully functional skin membrane, multiple printing inks are commonly used for composite inkjet printing and are combined with “bio-paper” for layer-by-layer printing. The construction of a clinically applicable multilayered and complex skin membrane has always been a challenge for 3D skin membrane technology. Miguel et al. [89] constructed an asymmetry multilayered asymmetric skin membrane, which is more suitable for treating patients with skin injuries, using composite 3D printing technology and electrospinning technology. Therefore, to create a skin membrane that meets practical needs, multiple manufacturing technologies need to be combined to produce complex membrane structures that meet the required specifications.

#### 2.3.2. Serosal Membranes

Serosal membranes include peritoneum, pleura, amniotic membrane, and pericardium [90]. According to the difficulty with producing this type of membrane outside the body and then implanting it, they are often produced inside the body. In situ 3D printing is a promising approach to address this challenge. Zhao et al. [91] proposed a new manufacturing strategy using in situ 3D printing technology during minimally invasive surgery assisted by seven-axis robots to achieve small-scale in situ 3D printing of amniotic membranes. They demonstrated the feasibility of this approach via animal experiments, providing a new solution for the in situ manufacturing of internal membranes.

#### 2.3.3. Tubular Tissue Membranes

Tubular tissue membranes include the cardiovascular membrane [92], esophageal membrane [93], tracheal membrane, intestinal membrane, and urethral membrane. These membranes are commonly used for tubular tissue regeneration [94], as well as pharmacological and toxicological studies [95]. The difficulty in building these membranes lies in constructing large-sized, multilayered, hollow tubular structures; hence, some additional support measures tend to be adopted in the process [96]. For example, some commonly used support measures include hydrogel-assisted suspension printing and sacrificial material-assisted printing [97].

#### 2.3.4. Connective Tissue Membranes

Connective tissue membranes include the periosteum [98], fascia, and synovium membrane. This type of membrane is closely related to the induction of bone [99,100], cartilage tissue [101], and muscle tissue [102] regeneration. A major challenge with this type of membrane is to maintain the original mechanical properties of the tissue after implantation. Larson et al. [103] modified the structure of the microextrusion printing nozzle to achieve the printing of multi-material helical structures with high toughness and elasticity, which were highly matched to muscle tissue function. Wang et al. [104] achieved a type of biomimetic stretchable nanofiber yarn scaffold, which was implanted into mouse tendon tissue and found to help the mouse perform moderate exercise following the repair of tendon defects. The above two examples demonstrate that the mechanical properties of 3D membrane structures can be improved by changing the morphology of the extruded microfilament in microextrusion 3D printing, thereby maintaining the original mechanical properties of the tissue after implantation.

#### 2.3.5. Other Tissue Membranes

Tympanic membranes [105], corneas [106], retinas [107], and other membranes can also be personalized and manufactured using multilayered 3D printing with various biomaterials to meet the clinical demand for tissue regeneration.

### 2.4. Limitations of 3D Printing

The existing 3D printing technology can achieve complex structural construction and the personalized design of various tissue membranes, but its application in more complex membrane structures is limited by issues such as printing precision, multi-material printing, and composite printing.

Additionally, the existing 3D printing membranes lack feedback mechanisms from biological signals, making it difficult for physicians to detect the repair status of the defective site in real time and to dynamically develop treatment strategies for patients. However, the recent development of sensors has brought possibilities for the construction of intelligent 3D membranes.

## 3. Sensors

**Figure 3:**
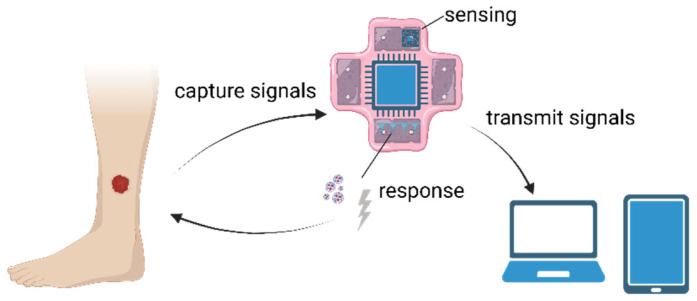
Common working principle of 3D membrane-binding sensors. A regenerative sensor captures signals from its recognition elements and may use its signal conversion elements to transmit signals to outer devices (smartphones or computers). Some responsive elements may respond to the wound spontaneously by releasing drugs or providing electrical stimulations to propel regenerative progressions. Created with BioRender.com (accessed on 21 August 2023).

A sensor is defined as an entity that retrieves the state of the sensed object and then pushes the collected data to one central processing and/or storage unit. Sensors can be divided into physical or virtual sensors [108]. However, 3D membrane-binding sensors are a type of sensor that combines the functions of physical and virtual sensors and may provide regenerative signals and feedforward spontaneous signals to respond to wounded tissues and organs. It is composed of molecular recognition elements (living animal and plant slices), corresponding signal conversion elements, and responsive elements. Figure 3 shows the common working principle of 3D membrane-binding sensors.

**Figure 4:**
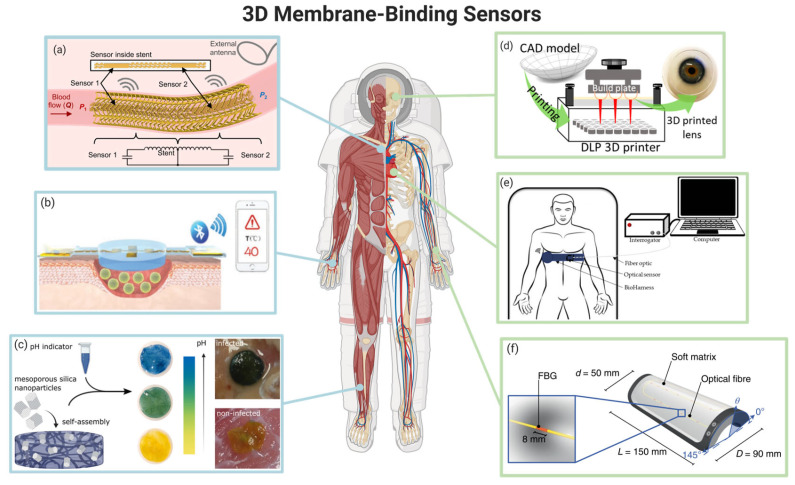
Examples of 3D membrane-binding sensors. (**a**) Fully implantable vascular membranes with printed soft sensors [114]. (**b**) Smart flexible electronic-integrated wound membranes for real-time monitoring and on-demand treatment of infected wounds [115]. (**c**) pH-responsive nanocomposite wound dressings [116]. (**d**) Smart contact lenses for continual glucose detection [117]. (**e**) 3D membrane-binding sensors for respiratory rate (RR) and heart rate (HR) monitoring [118]. (**f**) Artificial skin integrating transducers [110]. Created with BioRender.com (accessed on 21 August 2023).

Over the past decades, integrated with 3D regenerative membranes, the functions of 3D membrane-binding sensors have varied from time to time. Traditional sensors monitor wound conditions but are usually passive to signals they capture, while novel sensors are a type of sensor that may generate an active response to the wound sites. Moreover, binding with artificial skin, sensor-binding regenerative 3D membranes enable large-area tactile-sensitive skin production of a possible substrate of tissue regeneration and provides chronic wound care management [12,109,110,111]. In cardiac tissues, constructing a tissue-sensor platform with 3D printing technology can provide real-time and continuous monitoring of the physiological condition of the heart, so as to assist tissue regeneration [112]. In the human nervous system, the use of electrical signals as 3D membrane-binding sensors for repairing impaired neural tissue open up a new avenue of thinking about nerve regeneration therapy [113]. Figure 4 shows the various uses of 3D membrane-binding sensors.

### 3.1. Traditional Sensors for 3D Membranes

3D membrane-binding sensors can aid in monitoring and controlling wound infections. After using 3D printing technology to construct the scaffold, shortening the treatment time and providing solutions to control the progression and healing of wounds are very important [119]. With the development of a 3D wound-measuring camera to provide information on the wound area and the phase of wound healing, more attention has been paid to 3D membrane-binding sensors due to their provision of deeper and more detailed information on regenerative progression [120].

Former studies have shown that 3D-printed wound dressings contain a variety of microelectronic sensors for the real-time monitoring of the wound environment, which can send out signals to the clinician to report information such as graft failure or complications. These types of traditional regenerative smart 3D membranes incorporate pH, temperature, and oxygen sensors. Most of these traditional sensors are passive, with limited active responses in wound healing.

#### 3.1.1. Traditional Sensors for Regenerative Skin Membranes

pH-sensing 3D membranes: The pH of intact and non-infected skin is slightly acidic and typically varies between 4 and 6, while that of chronic wounds is typically in the range of 7 to 9. Hence, enclosing pH sensors into wound dressings has the potential to provide an assessment of the wound status, facilitating the detection of early-stage infections [121]. New studies are focusing on optimizing the sensitivity and response time of such sensors.

Temperature Sensor—Integrated Artificial Membranes: Injuries independently and interactively influence deep body temperature [122]. Upon the integration with hydrogel membranes, temperature sensors can continuously collect wound temperatures and detect bacterial infection, transmitting to the smart phone in real time and providing effective treatment based on clinical needs [115]. The trend of change in the wound temperature clearly provides doctors with the alteration in the wound stage, giving them a convinced clue of the wound recovery condition.

Oxygen—Sensing Membranes: Oxygen is a critical component in many biological processes and is essential for wound healing. Chronic wounds are typically characterized as being hypoxic in that the partial pressure of oxygen (pO_2_) in the center of the wound is often below a critical threshold that is necessary to fully support the enzymatic processes necessary for tissue repair, stressing the importance of real-time monitoring of the oxygen concentration in wound areas [123]. Regarding the wound healing models, Roussakis et al. [124] developed a collagen–dextran oxygen-sensing bio-composite scaffold membrane in which a phosphorescent oxygen sensor was incorporated to monitor the physiological oxygen consumption in vivo and provide an assessment of tissue oxygenation during wound healing.

Protein—Sensing Membranes: Protein concentrations in wounds have been used as an indicator of the state of the wound stages due to the stability of protein concentrations toward the active external environment surrounding the exudate [125]. El Saboni et al. [126] designed a flexible textile-based protein sensor that was embedded in wound dressings and was able to detect bovine serum albumin at concentrations ranging from 30 to 0.3 mg/mL, with a sensitivity of 0.0026 µA/M. Currently, to provide stressing indicators in wound healing, 3D membrane-binding sensors are being developed to integrate several detection indicators (e.g., pH, temperature, pO_2_, and protein concentrations) into one.

Metabolism Disease—Sensing Membranes: A regenerative sensor-binding 3D membrane may continuously monitor prevalent chronic metabolic disease. The traditional finger piercing glucose tests of diabetes incur incentive pain during detection, representing one of the main barriers to daily blood glucose monitoring. Skin pricking at alternate sites that have fewer nerve endings than fingertips has been suggested as a means to increase blood glucose monitoring compliance at home [127,128]. Cui et al. [129] developed a microneedle biosensing device manufactured with 3D printing technology to monitor diabetes. Inserted into the dermis layer of the mouse skin, this 3D membrane showed an accurate sensing performance for monitoring subcutaneous glucose levels in normal and diabetic mice. This study revealed that 3D printing can be applied to accelerate the recovery of chronic disease. New achievements may be used in regenerative 3D membrane-coupled sensors to monitor other diseases associated with tissue regeneration, and thus, promote the development of disease detection techniques.

#### 3.1.2. Traditional Sensors for Other Usages

Blood Pressure—Sensing Membranes: Sensors also play an important role in the regeneration of other tissues and organs in vivo. Indeed, a previous study showed that a battery-less pressure sensor based on an LC circuit and coupled with a 3D-printed biodegradable polymeric smart stent could be integrated into a 3D-printed polymeric stent to provide the wireless monitoring of the pressure in a blood vessel to follow disease progression and treatment [114,130].

Other Special Organ—Sensing Membranes: 3D membrane-binding sensors also play an important role in the regeneration process of the heart, blood vessels, and bones. A previous study showed that a battery-less pressure sensor based on an LC circuit and coupled with a 3D-printed biodegradable polymeric smart stent could be integrated into a 3D-printed polymeric stent to provide the wireless monitoring of the pressure in a blood vessel to follow disease progression and treatment [114,130]. Polley et al. [131] developed a 3D-printed piezoelectric barium titanate-hydroxyapatite scaffold, which combined smart and additionally electrically active biomaterials to display piezoelectric values, and thus, improve bone regeneration.

### 3.2. Novel Sensors for 3D Membranes

Wound healing is a highly dynamic process that may take years to recover. Incorporating three overlapping phases encompassing inflammation, proliferation, and remodeling, it means that any disruption of the three phases may lead to abnormal wound healing [132]. Meticulous curation by physicians is unrealistic, especially in this long-term healing process; hence, as mentioned previously, integrating therapeutic molecules and electrotherapy with 3D membranes represent an effective strategy to resolve this issue [133]. Some novel 3D membrane-binding sensors that are integrated into wound dressings and artificial tissues or organs have the ability not only to sense and detect the wound environment conditions but also to give spontaneous feedback to wound sites, serving to keep the patient informed about their condition and reduce physician intervention to some extent [116,120,126,134]. Wound pH-responding sensors, flexible bioelectronic sensors, and flexible bio-implanted sensors are new types of 3D membrane-binding sensors that provide ideal integrations with the soft, curvilinear, and elastic tissues and the unique capability of multimodal functions, enabling the better monitoring of the wound healing status, as well as providing advanced wound care and a spontaneous stimulation to accelerate the healing status [134,135]. Most of these 3D membrane-binding sensors are active or partially active in responding.

#### 3.2.1. Novel Sensors for Regenerative Skin Membranes

pH—Responding 3D membranes: Based on the sensing function of pH sensors, recent studies have shown that those pH-responding membranes may release drugs at the wound site via the feedback of pH sensors. Akbari et al. [116] developed a multifunctional hydrogel-based wound dressing by mapping the pH of the wound using an array of printed sensors, which initiates the delivery of a drug-releasing scaffold to release antibacterial agents at the wound site. Moreover, a user-friendly interface was designed to display the results and record the pH values for the continuous monitoring of the wound condition. The data were uploaded on a secure cloud storage drive, which allowed medical personnel to access the patient data and monitor the wound condition in real time [136]. Mirani et al. [137] demonstrated the high regenerative efficacy of wound dressing by monitoring the infection and supporting wound healing via antibiotic and growth factor delivery investigations in mouse models. With the development of electronic information and image processing technology, binding pH sensors into regenerative 3D-printed membranes serves to elevate the treatment monitoring of wounded skin, which provides early detection of deteriorative chronic diseases and the release of regenerative drugs in real time [121]. By monitoring and providing effective responses to the healing stages, pH-responding sensors potentially give direct access to the wound status without disturbing the wound bed.

Electrotherapy Binding Sensors: Additionally, a wireless closed-loop smart bandage with integrated sensors and simulators can be used to enhance the incorporation of both the sensors and simulators of the current smart bandage by activating a spontaneous intervention to promote the healing of chronic wounds, as well as to reduce the chance of detachment of existing adhesive dressings, which may damage the delicate adhesions of natural tissues. To ensure an intimate skin interface and robust electrical communication between the circuit and skin, these sensors work through a soft layer of hydrogel (a substance similar to the 3D membrane). This type of conductive hydrogel electrode may mediate tissue adhesion and detachment by adjusting temperature changes. As an appendage of 3D regenerative membranes, this type of integrated multimodal sensor and simulator for real-time monitoring and active wound care treatment could be attributed to the activation of bioregenerative genes in the monocyte and macrophage cell populations, thus considerably minimizing the need for physician intervention [134].

Bioresorbable Inflammatory Controlling Sensors: In terms of more spontaneous stimulation-provided sensors, W. Song et al. [133] provided a new bioresorbable, wireless, and battery-free system, with the ability to track inflammatory responses by mimicking a naturally occurring stimulation process of endogenous electric fields to promote healing by applying electric fields to restore endogenous wound currents and recapitulate the natural healing mechanism. Evidence of the reduction in cytokines and interleukin-6 demonstrated the success of this sensor-based system. Notably, this system can transmit real-time monitoring by sending signals to smartphones. In addition, after the healing process is complete, bandages and electronics can dissolve harmlessly in the body. Unfortunately, this kind of sensor-based system is not yet integrated with 3D-printed wound scaffolds; nevertheless, it provides a viable way in which to accelerate the tissue regeneration process. Prior to this, Yin et al. [138] developed a 3D-printed microheater sensor-integrated drug-encapsulated microneedle patch system for pain management, providing a possible pathway to integrate bioresorbable sensors with 3D imprinting controlling systems.

#### 3.2.2. Novel Sensors for Regenerating Other Tissues and Organs

Wearable Sensors: Beyond implanted regenerative 3D membrane-binding electronic sensors, smart wearable sensors interconnected with advanced wound dressing bandages seem to provide a substitute or alternative solution for hard-to-heal wounds by minimizing the risk of disease infection [111]. Indeed, wearable sensors have been successfully applied in continuous glucose monitoring and neural network detection. 3D printing can be used in contact lenses [117], where, in combination with 3D membrane-binding sensors, it may be used to monitor tear glucose levels for diabetic diagnosis, as well as to trigger the release of drugs from reservoirs for treating diabetic retinopathy [139]. Additionally, a flexible artificial intelligence-guiding wearable sensor can be operated with a deep artificial neural network algorithm for chronic wound monitoring via a wound dressing-integrated bandage [111]. Flexible sensors are also core components of intelligent wearable technology in vitro, which can convert stress and strain into electrical signals, and thus, accurately monitor human body indicators in real time [140,141,142].

Printed Sensors: Some of the latest research on sensor development shows that sensors themselves can be 3D printed to integrate with artificial tissues built outside the body. Based on 3D printing technology and plasma processing, highly sensitive strain sensors have a wide application in tissue regeneration, including the ability to be attached to skin, muscles, heart, and other organs to monitor wrist pulses, muscle movements, and other human motions [143]. Some of the other 3D-printed sensors are based on fiber Bragg grating technology (FBG) for respiratory rate and heart rate monitoring, which make the regenerative vitro 3D membrane more intelligent and easier to produce [118]. Due to the simpleness, inexpensiveness, and high reproducibility of the sensor, it may acquire wider application in constructing alternative vitro 3D membranes. These types of FBG sensors or transductors may be applied in soft electronic skin for sensitizing large areas of robot bodies and enable human–robot cooperation with the combination of artificial intelligence [110]. Bernasconi et al. [144] developed a layer-by-layer fabrication of hydrogel microsystems for controlling drug delivery. Such sensors use steerable microrobots to facilitate the diffusion of chemicals from the hydrogel layers to the external environment, leading to a promising wound care.

### 3.3. Development and Applications of 3D Membrane-Binding Sensors

#### 3.3.1. Development of 3D Membrane-Binding Sensors

The Accuracy of 3D Membrane-Binding Sensors: Calibration is important for sensors, as it ensures the reliability of the detected data. The internal components of the sensor and the external environment will both affect the accuracy of its detection results, so the calibration of the sensor is quite crucial [145]. By measuring the artificial wound fluids with different pH values and comparing the results with a commercial pH meter, it may reduce the internal errors of sensors, and thus, improve the accuracy of membrane-binding pH sensors [12]. However, how to maintain the accuracy of the sensors upon transplantation is still a tough question. Researchers suggests that developing a particular algorithm to automatically adjust the collected signals may reduce these false reads [146]. In addition, for some wearable sensors, if they are based on a single sensing mechanism, their accuracy is quite susceptible to interference from environmental factors when contacting with other fluids, such as nonlinear friction and electrical disturbances. Therefore, the perception and measurement accuracy of these sensors are limited to some extent. Hence, the integration of multifunctional sensing schemes to achieve precise object discrimination has been added to address these problems [147]. Moreover, using deep learning algorithms to evaluate and calibrate wearable sensors during human activity may further improve the accuracy of sensors [110].

4D Printing: Responsive electronic sensors are considered to have value in realizing the bottleneck of 3D printing in detecting and responding to dynamic changes in tissue conformation during recovery, where responsive electronic sensors come into being [148,149]. This type of regenerative method can dynamically reshape in response to tissue regeneration in real time [150]. Adding the dimension of “time” to 3D printing, the concept of four-dimensional printing (4D printing) was first proposed by Skylar Tibbits. 4D printing is often defined as a fundamental application that explores both autonomic and non-autonomic systems with different stimuli, such as temperature, current, moisture, light, and sound, to facilitate the fabrication of complex functional biological architectures [151,152]. 4D printing has two main development directions in tissue regeneration fields; one is programing the codes of components and structures to create controllable changes, and the other is using shape memory materials via 3D printing manufacturing [153,154]. The former corresponds to the sensor-responsive 3D membrane technology mentioned in this review, while the latter, just as Lai et al. [155] investigated, provides a strategy for fabricating porous scaffolds to facilitate the self-folding ability and the controlled release of growth factors in scaffold applications. Miao et al. [156] concluded that 4D printing is a good candidate that may significantly advance the development of biomedical scaffolds with advanced 3D fabrication techniques.

In conclusion, 4D-printed membranes show great potential in adapting to the dynamic structure of human tissues as well as in responding to specific external or physiological conditions. 4D printing for biomedical applications is an emerging research field that has already demonstrated its outstanding potential for the future development of the next-generation technique of the construction of regenerative and responsive 3D membranes [104].

#### 3.3.2. Applications in Skin

Sensor-binding 3D regenerative membranes are widely used in clinical research. Skin contributes critically to health via its role as a barrier tissue against a multitude of external pathogens [157]. Hence, the demand for skin biofabrication is still rising with great speed. In this section, we discuss various types of novel applications incorporating disease modeling, electronic skin, organ-on-a-chip, and drug development.

Disease Modeling: The construction of 3D membranes enables the production of multicellular tissue models as assay platforms for drug screening. Liu et al. [158] developed an artificial atopic dermatitis (AD) disease-like tissue using a 3D membrane, fabricating skin equivalent tissues of varying physiological complexity, including human epidermis and non-vascularized and vascularized full-thickness skin tissue equivalents, in a multi-well platform to enable drug screening. Additionally, as mentioned previously, the integration of smart sensors based on excellent simulated disease engineering scaffolds facilitates the detection of disease simulation effects and provides real-time feedback on disease regeneration progression. The detectable 3D membrane is helpful to judge the quality of the disease model, while the responsive 3D membrane is conducive to the development of treatment methods for the self-treatment of diseases, representing an important contribution to the intelligence of future medical treatment.

Drug Development: With the appeal of banning animal testing for cosmetic purposes and the intention to reduce animal testing in clinical research, 3D membranes may serve as an animal substitute due to their similarity to human skin and organs. Therefore, 3D membranes may be considered an appropriate platform to perform the assessment and screening of cosmetic and pharmaceutical formulations [31]. Moreover, constructing artificial 3D membranes is often cheaper and more representative of the physiology or structure of human skin in modeling skin wounds, taking the place of animal models in demonstrating the pharmacological effects of a drug [159,160]. Lukács et al. [161] developed microfluidics for artificial 3D membranes, a “skin-on-a-chip model”, which was integrated with microfabricated sensors and aimed to develop proper drug formulations and optimize the delivery of their active ingredients via the dermal barrier.

#### 3.3.3. Applications in Other Tissues and Organs

Organ-on-a-Chip: Microfluidics technology may also be used in cardiovascular, kidney, and brain organoids. Organoids are the in vitro miniaturized and simplified model systems of organs. Due to their exceptional ability to recreate precise cellular organizations, 3D organotypic models facilitate the investigation of the interactions between different sub-tissue level components by providing physiologically relevant microenvironments for cells in vitro [158,162]. Using sensor-binding 3D membranes, the “organ-on-a-chip” system can integrate 3D membranes and organotypic culture. This type of sensor-binding 3D membrane is termed a “microfluidic membrane”; with the application of the microfluidic membrane, organ-on-a-chip system modeling may allow for the recreation of the tumor microenvironment (cancer-on-a-chip) and the modeling of immune organs (bone marrow-on-a-chip), enhancing the success rate of drug development [163].

## 4. Conclusions and Perspectives

### 4.1. Advantages and Limitations

Due to the 3D printing technique, 3D regenerative membranes provide a repeatable method in tissue regeneration to some extent; however, there remain some limitations in sensor-binding 3D regenerative membranes, as listed in Table 2.

Therefore, these limitations should be fully considered when designing 3D sensor-binding membranes. Using biodegradable and biocompatible materials (e.g., zinc, molybdenum, and biomaterials) to obtain a 3D sensor-binding membrane is a crucial factor to avoid rejection, inflammation, and tissue dysfunction in vivo [164]. To apply 3D sensor-binding membranes in actual clinical use, they may meet strict regulations set by governments. According to the Food and Drug Administration’s regulations, 3D sensor-binding membranes are Class III devices. Therefore, it is necessary to verify the stability, safety, and reliability of functionality before clinical use, so as to meet the application standards.

### 4.2. Conclusions and Prospectives

With the development of 3D printing and sensor technologies, 3D membrane-binding sensors for various tissues have become increasingly mature and suitable for clinical practice. Regenerative multilayered 3D membranes can be produced by combining multi-suitable printing inks with various 3D printing methods. Moreover, 3D membranes can be printed in situ within the body by using surgical robots and appropriate 3D printing technologies. Additionally, by manipulating the shape of the extrusion nozzle, unique fiber deposits can improve the mechanical performance of the 3D membrane while also helping to induce tissue regeneration and restore tissue function to a certain extent. These recent advances in 3D membrane biomanufacturing enable future 3D membranes to be constructed in situ within the body to improve tissue function while ensuring the retention of material tissue regeneration induction properties. However, these manufactured membranes still lack signal response mechanisms and cannot adapt to the changes in the patient’s internal environment and the tissue regeneration process. As a result, incorporating sensors into 3D membranes has become a great prospect for future development.

Currently, sensor technology has been used in 3D membranes optimizing the procedure of wound regeneration. Sensors capture signals from their recognition elements to collect changes in biological signals such as pH, temperature, oxygen pressure, and muscle movement. Traditional sensors may meet the needs of monitoring wound conditions, while novel sensors provide spontaneous feedback signals and adjust treatment plans accordingly. Alternatively, feedback to the internal response section of the 3D membrane system can achieve the self-intelligent adjustment of the 3D membrane system. Internal response mechanisms for 3D membrane systems currently include controlling changes in membrane properties, controlling sensing robots, and foldable systems or biological factor sustained-release systems fabricated via 4D printing, all of which achieve smart integration after incorporating the sensors into 3D membranes. Hence, integrated with 3D membrane-binding sensors, 3D membranes acquired the ability of monitoring tissue regeneration as well as inducing tissue regeneration.

Relevant studies have shown that molecules with multiple slow-release properties (e.g., hydrogen) can greatly promote the regeneration of skin and other tissues [165]. In the future, 3D scaffolds using hydrogen molecules as slow-release materials may be used to improve the functionality of 3D membranes. We believe that 3D membrane technology can achieve greater flexibility to regenerate more tissues and organs. Indeed, 3D membrane technology developments are becoming more in-depth and detailed, allowing for the better stimulation of nerves and blood vessels in tissues to promote regeneration. In the future, research should focus on making these microelectronic sensors more miniaturized, intelligent, and degradable, so that the combination of 3D membranes and electronic components can be more closely attached. Despite the difficulties in construction, the further development of 3D membranes is likely to lead to their extensive use in the clinic, as well as in disease modeling and drug developments, ultimately becoming an integral component of human medical treatment.

## Figures and Tables

**Table 1:** Common types of biomaterials used in printing inks.

Type	Biomaterials	Crosslinking	3D Printing Methods	Refs.
Animal-sourced natural ECM materials	Collagen	Thermal	Extrusion-based, droplet-based, laser-assisted	[64,65,66]
	Fibrinogen	Enzymatic	Extrusion-based, droplet-based	[67]
	Hyaluronan	Photic, enzymatic	Extrusion-based	[68]
	Decellularized extracellular matrix (dECM)	Photic, thermal, pH	Extrusion-based, droplet-based	[69]
	Gelatin	Photic, thermal, enzymatic, ionic	Extrusion-based, droplet-based, stereolithography-based	[70]
Non-animal-derived natural hydrogels	Cellulose	Photic, thermal, enzymatic, ionic, pH	Extrusion-based, droplet-based	[71,72]
	Alginate	Photic, thermal, ionic	Extrusion-based, droplet-based, laser-assisted	[73]
	Chitosan	Thermal, enzymatic, ionic, pH	Extrusion-based	[74]
	Agarose	Thermal	Extrusion-based, droplet-based	[59,75]
	Carrageenan	Photic, thermal, ionic	Extrusion-based	[76,77]
Synthetic hydrogels	Polyethylene glycol (PEG)	Photic	Extrusion-based, droplet-based, stereolithography-based	[59,78]
	Polyurethane (PU)	Thermal	Extrusion-based	[79,80]
	Polyvinyl alcohol (PVA)	Chemic, ionic	Extrusion-based	[81,82]
	Polylactic acid (PLA)	Thermal	Extrusion-based	[83]
	Pluronic F127 (PF127)	Thermal	Extrusion-based	[84]

**Table 2:** Advantages and limitations of regenerative 3D membrane-binding sensors.

Advantages	Limitations
High individualization, flexibility, and repeatability in manufacturing	Difficulty in selecting biocompatible materials
BN provides real-time monitoring and active wound care treatment with minimal physician intervention at wound sites	Biosafety: ethical issues and electronic reagents
Better regenerative effects	High costs and long healing times
Enables large-scale fabrication	Difficulty in remodeling blood vessels and nerve tissues

## Data Availability

Data presented in this study are available on request from the corresponding author.

## Abbreviations

3D	Three dimensional
4D	Four dimensional
dECM	Decellularized extracellular matrix
micro-CAL	Microscale computed axial lithography
pO_2_	Partial pressure of oxygen
AD	Atopic dermatitis
CAD	Computer-aided manufacturing
CAM	Computer-aided design
DMD	Digital micromirror device
ECM	Extracellular matrix
FBG	Fiber Bragg grating technology
GO	Graphene oxide
MI	Myocardial infarction
PEG	Polyethylene glyco
PF127	Pluronic F127
PLA	Polylactic acid
PU	Polyurethane
PVA	Polyvinyl alcohol
TPP	Two-photon polymerization
